# Poor receptive joint attention skills are associated with atypical gray matter asymmetry in the posterior superior temporal gyrus of chimpanzees (*Pan troglodytes*)

**DOI:** 10.3389/fpsyg.2014.00007

**Published:** 2014-01-29

**Authors:** William D. Hopkins, Maria Misiura, Lisa A. Reamer, Jennifer A. Schaeffer, Mary C. Mareno, Steven J. Schapiro

**Affiliations:** ^1^Neuroscience Institute, Georgia State UniversityAtlanta, GA, USA; ^2^Language Research Center, Georgia State University Atlanta, GA, USA; ^3^Division of Developmental and Cognitive Neuroscience, Yerkes National Primate Research CenterAtlanta, GA, USA; ^4^Department of Psychology, Agnes Scott CollegeDecatur, GA, USA; ^5^Department of Veterinary Sciences, The University of Texas MD Anderson Cancer CenterBastrop, TX, USA; ^6^Department of Experimental Medicine, University of CopenhagenCopenhagen, Denmark

**Keywords:** joint attention, chimpanzees, superior temporal gyrus, brain asymmetry in cognition, brain development

## Abstract

Clinical and experimental data have implicated the posterior superior temporal gyrus as an important cortical region in the processing of socially relevant stimuli such as gaze following, eye direction, and head orientation. Gaze following and responding to different socio-communicative signals is an important and highly adaptive skill in primates, including humans. Here, we examined whether individual differences in responding to socio-communicative cues was associated with variation in either gray matter (GM) volume and asymmetry in a sample of chimpanzees. Magnetic resonance image scans and behavioral data on receptive joint attention (RJA) was obtained from a sample of 191 chimpanzees. We found that chimpanzees that performed poorly on the RJA task had less GM in the right compared to left hemisphere in the posterior but not anterior superior temporal gyrus. We further found that middle-aged and elderly chimpanzee performed more poorly on the RJA task and had significantly less GM than young-adult and sub-adult chimpanzees. The results are consistent with previous studies implicating the posterior temporal gyrus in the processing of socially relevant information.

At approximately 6–8 months of age, typically developing children begin to respond to a number of non-verbal socio-communicative cues, including gaze, pointing and verbal bids (Adamson, 1996; Flom et al., 2006; Leavens, 2012). These are sometimes referred to as receptive joint attention (RJA) skills. Individual differences in RJA skill have been linked to the subsequent development of early linguistic skills, including comprehension and production of language, as well as other cognitive abilities, such as imitation learning and theory of mind (Mount et al., 1989; Charman et al., 2000; Slaughter and McConnell, 2003). For example, a number of studies have shown that the age of onset of both the initiation of, and response to, joint attention cues predicts the rate of language development in typically developing children (Bates et al., 1975, 1987; Carpenter et al., 1998; Morales et al., 2000; Nichols et al., 2005; Whalen et al., 2006; Mundy et al., 2007; Brooks and Meltzoff, 2008).

Not only is RJA a universal trait in typically developing children, there is also evidence for its existence in great apes and other primates, suggesting it has a long evolutionary history. Studies in a number of laboratories have shown that Old and New World monkeys and apes will not only follow gaze (Brauer et al., 2005; Rosati and Hare, 2009), but can follow gaze around barriers, and follow manual pointing gestures to specific locations (Tomasello et al., 1999; Brauer et al., 2005; Amici et al., 2009). As with human infants (Moll and Tomasello, 2004), there are considerable individual differences in gaze following and RJA performance in nonhuman primates. For instance, Russell et al. (2011) examined, among a number of measures, gaze following on three trials in a sample of 83 chimpanzees. Fifteen percent of chimpanzees failed to follow gaze on all three trials, whereas 41% successfully followed gaze on all three trials. Herrmann et al. (2007), 2010 have reported similar individual differences in gaze following and comprehension of pointing responses in chimpanzees and bonobos.

Though the cognitive abilities of primates to respond to different socio-communicative cues are well documented, our understanding of the neural mechanisms underlying their expression are poorly understood. In the current study, we examined whether individual differences in RJA performance are linked to variation in the volume or asymmetry of the posterior superior temporal gyrus (p_STG) in chimpanzees. We focused on the p_STG as the cortical region of interest for several reasons. First, in Old World monkeys, single cell recording and reversible lesion studies have shown that neurons within the superior temporal gyrus and sulcus respond to certain social cues, such as eye gaze (Emery, 2000; Kamphius et al., 2009; Shepherd, 2010; Roy et al., 2012), and these results are consistent with fMRI findings in humans (Williams et al., 2005; Itier and Batty, 2009). Second, atypical patterns of asymmetry in the p_STG have been described in clinical populations in which deficits in social cognition and perception are prominent endophenotypes, notably schizophrenia (Barta et al., 1997; Klar, 1999; Kwon et al., 1999; Hirayasu et al., 2000; Sommer et al., 2001; Dollfus et al., 2005) and autism spectrum disorder (ASD; Zilbovicius et al., 2006; Jou et al., 2010; Chen et al., 2011). Third, in a recent review, Mundy and Newell (2007) proposed that responding to joint attention is associated with regions in the posterior superior temporal lobe and portions of the parietal lobe. For instance, in human adults, Williams et al. (2005) performed fMRI on subjects when they were engaged in joint attention compared to non-joint attention processing and found a significant number of brain regions active, including the ventromedial left prefrontal cortex (BA44, BA45), superior temporal gyrus (BA22), superior frontal cortex (BA10) anterior cingulate cortex (BA24), and regions within the basal ganglia (putamen and caudate). In terms of preverbal infants, far less is known, but studies employing scalp recording methods, such as EEG and ERPs, have reported significantly greater activity in posterior temporal and parietal regions when responding to joint attention cues (Mundy et al., 2000). These collective findings led us to focus on the p_STG as a targeted region potentially associated with RJA performance.

Chimpanzees are particularly valuable model species for understanding the neurobiology of social cognition for several reasons. First, as noted above, they have well developed RJA skills and, like humans, their responses to different socio-communicative cues fall along a continuum. This study was designed to delineate several points on this continuum that might be useful for understanding human social cognition as it relates to different clinical population such as schizophrenia and ASD. Second, anatomically and cytoarchitectonically, there is considerable homology between the human and chimpanzee brain (Hopkins and Nir, 2010; Spocter et al., 2010; Hopkins, 2013). For instance, the sulcal landmarks used to quantify the planum temporale and planum parietale in humans and chimpanzees are nearly identical (Hopkins and Nir, 2010; Gilissen and Hopkins, 2013) and, like humans, chimpanzees show leftward asymmetries in these regions, which are not found in other nonhuman primate species (Gannon et al., 2008; Lyn et al., 2011).

To test the hypothesis of the role of p_STG in RJA proposed by Mundy and Newell (2007), we measured RJA skills in chimpanzees on a task developed by Dawson et al. (2002), previously employed with typically developing children, as well as those at risk for autism. We also quantified the gray matter (GM) volumes of the anterior and posterior, superior temporal gryus (STG) in these same chimpanzees. We hypothesized that if variation in RJA skills is associated with cortical organization within the STG, then significant differences would be found between chimpanzees that perform poorly compared to those who perform moderately or very well on this task. Based on previous results from structural and functional imaging studies, we further hypothesized that associations between GM volume and/or asymmetry would be specific to the posterior, but not anterior, region of the STG.

## MATERIALS AND METHODS

### SUBJECTS

Subjects for this study included 191 captive chimpanzees (*Pan troglodytes*) housed at either The University of Texas MD Anderson Cancer Center (UTMDACC) or the Yerkes National Primate Research Center (YNPRC) of Emory University. There were 114 females and 77 males housed in social groups that ranged in size from 2 to 13 individuals. The chimpanzees ranged in age from 8 to 53 years (Mean = 26.24, s.d. = 10.68). Based on the age range, we classified our chimpanzee sample into four age groups including sub-adult (8–16 years), young-adult (17–25), middle-aged (26–39 years) and elderly (40 years or older). Based on these cut points, there were 31 sub-adult, 75 young-adult, 55 middle-aged, and 30 elderly chimpanzees in the sample. The age groups cut-points were adopted from previous studies in captive chimpanzees (Herndon, 2009; Lacreuse et al., 2014). Subjects had access to both indoor and outdoor enclosures throughout the day and night, and participation in the study task was voluntary. All procedures were approved by the local Institutional Animal Care and Use Committees and followed the Institute of Medicine guidelines for use of chimpanzees in research.

### PROCEDURE

#### Receptive joint attention

**FIGURE 1:**
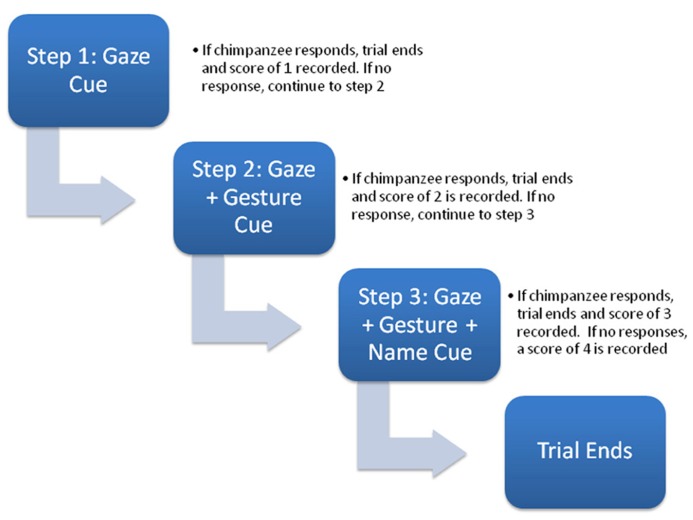
** Schematic diagram of the sequence of socio-communicative cues presented during each trial (see text for description)**.

The task used to measure RJA was identical to one developed by Dawson et al. (2002) in human children. Experiments were conducted with subjects either independent of their social group or divided into subgroups of two or three individuals, where the non-focal animals did not distract or interfere with the testing of the focal subject. Each subject received four test trials and a diagram of the trial procedure is shown in **Figure 1**. The goal of the task was to assess the number of social cues needed to elicit an orienting response from the subject. To accomplish this, each trial consisted of three hierarchical steps with an increasing number of social cues provided to the subjects in order to elicit an orienting response.

At the onset of testing for each trial, the focal chimpanzee would sit calmly in front of the experimenter they would engage them in some type of husbandry behavior. This might include the chimpanzee showing their foot, hand, arm or some other body part for inspection. When the chimpanzee was compliant with these requests, it was given small pieces of food. When the experimenter sensed that the chimpanzee was socially engaged with them, they would stop interacting with them and look over their head for 5 s, then return to a neutral position and wait 5 s (Step 1). If the chimpanzee overtly oriented or looked back to where the experimenter had looked either during the cue or the 5-s following the trial was over and the subject was given a score of 1. If the chimpanzee failed to look during the 10-s response window in Step 1, the experimenter would re-engage the chimpanzee in the husbandry-type activities again until she again felt as though the subject was socially engaged. At this point, the experimenter would look over the subject’s head again and this time point with an extended arm/finger toward an imaginary object behind them for 5 s (Step 2). After this, the experimenter returned to her sitting position and waited an additional 5 s for the chimpanzee to respond. If the chimpanzee oriented or looked back to where the experimenter had looked and pointed during the 10-s response window, the trial was over and the subject was given a score of 2. If the focal chimpanzee failed to look during the response window in Step 2, as before, the experimenter re-engaged the chimpanzee in the husbandry-type activities. The experimenter, then again, looked over the subject’s head, pointed with an extended arm/finger toward an imaginary object behind them and said the chimpanzee’s name two times (Step 3). The experimenter then returned to her neutral sitting position and waited 5 s for the chimpanzees to respond. If the chimpanzee oriented or looked back to where the experimenter had indicated during the 10-s response window, the trial was over and the subject was given a score of 3. If the chimpanzee failed to respond at the end of Step 3, it was given a score of 4. To characterize the performance of the chimpanzees, we derived a composite overall score that reflected the average number of cues they needed to respond. For this variable, the score of each trial was summed across trials and divided by the number of trials (4; Mean_RJA). Higher Mean_RJA indicated that subjects needed, on average, more social cues to elicit an orienting response across all trials.

### MAGNETIC RESONANCE IMAGE COLLECTION

All chimpanzees were scanned during their annual physical examination. Magnetic resonance image (MRI) scans followed standard procedures at the YNPRC and UTMDACC and were designed to minimize stress. Thus, the animals were first sedated with ketamine (10 mg/kg) or telazol (3–5 mg/kg) and were subsequently anesthetized with propofol (40–60 mg/kg/h). They were then transported to the MRI scanning facility and placed in a supine position in the scanner with their head in a human-head coil. Upon completion of the MRI, chimpanzees were briefly singly housed for 2–24 h to permit close monitoring and safe recovery from the anesthesia prior to return to their home social group. All procedures were approved by the Institutional Animal Care and Use Committees at YNPRC and UTMDACC and also followed the guidelines of the Institute of Medicine on the use of chimpanzees in research. Fifty-seven chimpanzees were scanned using a 3.0 Tesla scanner (Siemens Trio, Siemens Medical Solutions USA, Inc., Malvern, PA, USA). T1-weighted images were collected using a three-dimensional gradient echo sequence (pulse repetition = 2300 ms, echo time = 4.4 ms, number of signals averaged = 3, matrix size = 320 × 320, with 0.6 × 0.6 × 0.6 resolution). The remaining 134 chimpanzees were scanned using a 1.5T G.E. echo-speed Horizon LX MR scanner (GE Medical Systems, Milwaukee, WI, USA). T1-weighted images were collected in the transverse plane using a gradient echo protocol (pulse repetition = 19.0 ms, echo time = 8.5 ms, number of signals averaged = 8, matrix size = 256 × 256, with 0.7 × 0.7 × 1.2 resolution).

### REGION OF INTEREST

**FIGURE 2:**
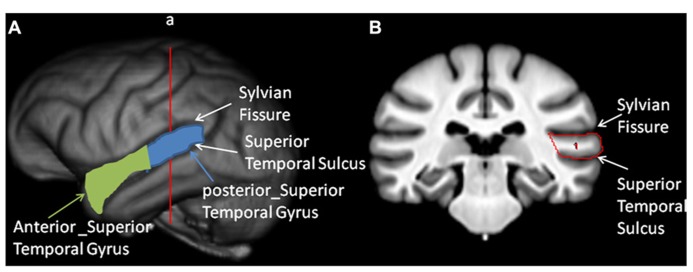
** Tracing of the anterior and posterior temporal lobe. ** Left panel: **(A)** 3-D reconstruction of the chimpanzee brain with the sylvian fissure (SF) and superior temporal sulcus (STS) labeled. Also, in green and light blue colors, the anterior and posterior superior temporal gyrus are outlined. Right panel: **(B)** Coronal view of the sulcl landmarks used to trace the superior temporal gyrus on a T-1 weighted MRI scan.

Prior to quantification of the anterior (a_STG) and posterior superior temporal gyrus (p_STG), all T1-weighted MRI scans were realigned in the AC–PC plane, skull-stripped and segmented into GM, white matter and Cerebral spinal fluid following procedures that have been described in detail elsewhere (Zhang et al., 2001; Smith et al., 2004). The superior temporal gyrus (STG) was primarily quantified in the coronal plane but, when necessary, the landmarks could be viewed simultaneously in the axial or sagittal plane using ANALYZE 11.0 software. The superior border of the STG was the sylvian fissure; the inferior border was the superior temporal sulcus and the lateral border was the surface of the temporal lobe (see **Figure 2**). Beginning at the temporal pole in each hemisphere, an object map was drawn around the gyrus using the landmarks described above. Moving posteriorly in 1 mm increments, the object maps were drawn on each image and continued until the sylvian fissure or superior temporal sulcus terminated. In some cases, the posterior sylvian fissure bifurcated into an ascending and descending branch, and we always followed the descending ramus as the superior border of the STG. To divide the STG into anterior and posterior regions, the total length of the gyrus, which corresponded to the number of images on which an object map was drawn, was determined and the median slice was identified. Images lower or equal to the median were defined as the a_STG region and images higher than the median were defined as the p_STG. The median slice was typically found at or about the anterior location of Heschl’s gyrus (HG). The object maps for each subject and hemisphere were saved. To calculate the GM volume of the a_STG and p_STG, the object maps that were traced on the T1-weighted scan for each hemisphere and region were applied to the segmented GM volume (see **Figure 2B**). The left and right hemisphere volumes (mm^3^) were computed by summing all the voxels found within the a_STG and p_STG object maps. All the images were traced by a single individual (MM) and prior to data collection, intrarater agreement was established using intraclass correlation coefficients within a sample of 10 individual brains. Intraclass correlations were positive and significant for both the left (*r* = 0.922, *p* < 0.01) and right (*r* = 0.972, *p *< 0.05) hemispheres. The person (MM) tracing the brains was blind to the sex and individual performance of the chimpanzees on the RJA task.

### DATA ANALYSIS

For each subject, we computed a percentage of GM volume by dividing the a_STG and p_STG GM values by the total GM volumes within each hemisphere. This was done to adjust for potential individual differences in total GM independent of the regions of interest. The percentage scores were averaged between the two hemispheres to create an overall estimate of GM for each region. In addition, we also computed asymmetry quotients (AQ) for GM within each region (GM_AQ_Ant, GM_AQ_Post). AQ scores were computed following the formula: [AQ = (R – L)/((R + L) × 5)] where R and L represent the respective GM percentages for the right and left hemispheres. Positive AQ values reflect right hemisphere biases and negative values reflect leftward asymmetries. The absolute value of the AQ indicates the strength or magnitude of the asymmetry. All analyses were performed using inferential statistics with alpha set to *p *< 0.05. *Post hoc* analyses, when necessary, were conducted using Tukey’s Honest Significant Difference test.

## RESULTS

### RECEPTIVE JOINT ATTENTION

In the initial analyses, we tested for sex and age effects on the Mean_RJA performance. For this analysis, we used analysis of variance with sex and age group as the between group factors, while the Mean_RJA scores were the dependent measure. We found a significant main effect for sex *F*(1,183) = 4.288, *p *< 0.04 and a significant interaction between sex and age group *F*(3,183) = 4.364, *p *< 0.006. The mean Mean_RJA performance for males and females from each age group are shown in **Table 1**. *Post hoc* analysis indicated that elderly females did significantly worse than middle-aged, young-adult and sub-adult females. For males, elderly and middle-aged individuals did significantly worse than young-adult and sub-adult apes.

**Table 1:** Average Mean_RJA and percentage GM volumes (+SE) for male and female chimpanzees in each age group.

	Age groups			
	Sub-adult	Young-adult	Middle-aged	Elderly
**Mean_RJA**
Females	1.95	1.99	1.81	2.63
	(0.19)	(0.14)	(0.14)	(0.18)
Males	2.21	2.13	2.85	2.33
	(0.23)	(0.14)	(0.20)	(0.28)
Overall	2.08	2.06	2.33	2.48
	(0.15)	(0.09)	(0.12)	(0.16)
**Percentage GM volume**
Females	2.68	2.58	2.56	2.32
	(0.11)	(0.05)	(0.06)	(0.09)
Males	2.55	2.34	2.28	2.19
	(0.13)	(0.07)	(0.11)	(0.15)
Overall	2.62	2.46	2.42	2.26
	(0.08)	(0.05)	(0.07)	(0.09)

### STG VOLUME AND ASYMMETRY

We examined the effects of sex and age on STG volume and asymmetry. In the volumetric analysis, we used a mixed-model ANOVA with the standardized GM *z*-scores for the anterior and posterior STG serving as the repeated measure, while sex and age group were the between-group factors. This analysis revealed significant main effects for sex *F*(1,183) = 6.661, *p *< 0.02 and age *F*(3,183) = 2.837 *p *< 0.04. There was also a significant interaction between sex and temporal lobe region *F*(1,183) = 5.316, *p *< 0.03. For the age main effect, *post hoc* analysis indicated that elderly chimpanzees had smaller GM volumes compared to sub-adult and young adult, but not middle-aged chimpanzees. The mean percentage GM volumes in each group are shown in **Table 1**. For the interaction between sex and temporal lobe region, *post hoc* analysis indicated no significant difference in GM volume for the a_STG region; however, for the p_STG region, males (Mean = 2.27, SE = 0.054) had relatively less GM than females (Mean = 2.56, SE= 0.054). No other significant main effects or interactions were found.

For asymmetries in the a_STG and p_STG, we also used a mixed model ANOVA with the AQ scores for each region serving as the repeated measure while sex and age group were the between group factors. A significant main effect for region was found *F*(1,183) = 27.624,* p *< 0.001. The mean AQ scores for the p_STG region (Mean = -0.080, SE = 0.013) were more leftward than the a_STG region (Mean = 0.023, SE = 0.013). Indeed, one sample *t *tests on the AQ scores revealed a significant population-level leftward bias for the p_STG *t*(190) = -7.214, *p *< 0.001, but no significant bias for the a_STG region *t*(190) = 0.709, *p* = 0.479.

### RELATIONSHIP BETWEEN MEAN_RJA AND STG VOLUME AND ASYMMETRY

In the next set of analyses, we integrated the measures of GM volume and asymmetry for the a_STG and p_STG regions into a series of partial correlation analyses as a means of predicting individual differences in RJA performance. Because we previously showed that age and sex influenced RJA performance, we sought to determine whether variation in either GM volume or asymmetry would account for a significant proportion of variability in performance over and above that of the variables of sex and age. Thus, we performed partial correlation coefficients between Mean_RJA performance and the a_STG and p_STG standardized GM volumes and AQ scores. The only significant partial *r*-value was between Mean_RJA performance and p_STG AQ scores (*beta* = 0.155, *p* < 0.04). Subjects with more rightward AQ scores showed poorer RJA performance.

### RELATIONSHIP BETWEEN GAZE PERFORMANCE ALONE AND STG VOLUME AND ASYMMETRY

**FIGURE 3:**
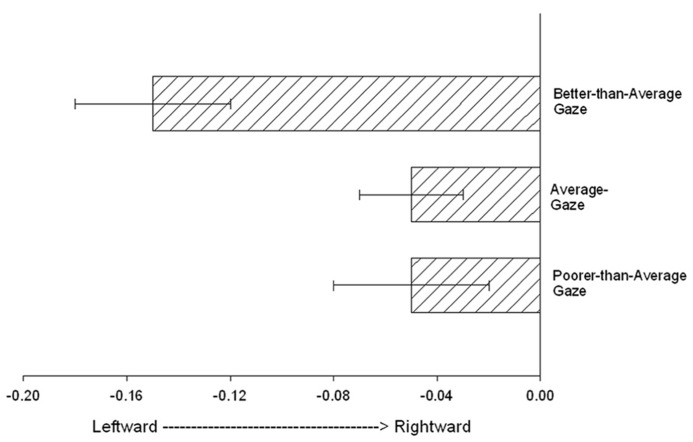
** The mean posterior superior temporal gyrus (p_STG) AQ scores (+/- standard error) for the Better-than-average_Gaze, Average_Gaze, Poorer-than-average_Gaze groups**.

The previous analyses focused on the association between Mean_RJA performance and variation in GM volume and asymmetry in the a_STG and p_STG regions. Because gaze following and response to gaze cues alone are important factors linked to variation in p_STG organization, we further examined whether performance on the gaze-following cue alone was associated with the neuroanatomical measures. For this analysis, we computed the number of trials on which the chimpanzees responded to the gaze cue alone. Scores could range from 0 to 4 (a 4 was recorded when the subject responded to gaze alone on all four trials). Based on these data, and in order to increase statistical power, we classified the chimpanzees into one of three groups, including poorer than average (score = 0, PTA_Gaze), average (score = 1 or 2, AVG_Gaze) or better than average (score of a 3 or 4, BTA_Gaze). We then compared the a_STG and p_STG volume and asymmetry scores between these groups as well as between sexes and age groups using analysis of variance. No significant main effects or interactions were found between gaze performance, sex and the a_STG and p_STG GM volume measures; however, for the AQ scores, we found a significant main effect for gaze performance on the p_STG scores *F*(2,167) = 4.054, *p *< 0.02. The mean p_STG AQ scores for the BTA_Gaze, AVG_Gaze, PTA_Gaze groups are shown in **Figure 3**. *Post hoc* analysis indicated that the mean p_STG AQ scores were significantly more leftward for the BTA_Gaze group compared to the PTA_Gaze group but did not differ from the AVG_Gaze group. No other significant differences were found. For the a_STG AQ scores, no significant main effects or interactions were found.

## DISCUSSION

The results of this study reveal several important findings. First, poorer performance on a task designed to assess RJA is associated with greater rightward asymmetries in the posterior, but not anterior, portion of the superior temporal gyrus. Second, we found significant age-related changes in performance on the RJA task and overall GM volume within the superior temporal gyrus. For the RJA task, older subjects performed more poorly than younger subjects. Further, the onset in decline on performance started at a younger age in males compared to females. For the GM volume, older subjects had lower percentages of GM compared to younger individuals.

With regard to the association between RJA and gaze performance and atypical asymmetries in the p_STG, our findings in chimpanzees are consistent with the hypothesis proposed by Mundy and Newell (2007), and are in general agreement with results in human clinical populations in which deficits in socio-communicative abilities are a significant endophenotype, such as schizophrenia (Sommer et al., 2001) or ASD (Boddaert et al., 2004; Zilbovicius et al., 2006). To be clear, we are not suggesting that our chimpanzees that respond poorly to socio-communicative cues are schizophrenic or autistic but rather that individual variation in RJA performance appears to be explicitly linked to asymmetries in the p_STG, but not the a_STG. We emphasize the word atypical asymmetry in this discussion because it is important to emphasize that the chimpanzees, as a group, show a leftward asymmetry in the GM volume of p_STG. Thus, individuals who fail to show a bias, or those with reversed asymmetries in the p_STg, are the ones who perform poorly on the RJA task.

The finding of a significant association between RJA task performance and atypical asymmetries in the p_STG also bears directly on theoretical and applied views of the role of brain asymmetries on individual fitness. A number of researchers have argued that having an asymmetrical brain confers some advantages from an evolutionary perspective (Ghirlanda and Vallortigara, 2004; Vallortigara and Rogers, 2005). With the context of the results reported here, it might be suggested that having an asymmetrical p_STG (and indeed, a leftward asymmetry) provides individuals with increased sensitivity for monitoring socio-communicative cues from conspecifics, such as gaze direction, head orientation and gestures. Many of these cues would be potentially important for selecting mates and/or avoiding conflict and agonistic encounters with conspecifics, and therefore afford some advantages to those individuals.

We also found age-related changes in both RJA performance and standardized GM volume. With respect to RJA, older chimpanzees performed more poorly than younger individuals. Similarly, older individuals had lower standardized GM volumes than younger individuals (see **Figure 2**). There is very little data on age-related changes in cognition and cortical organization in chimpanzees, but the findings reported here are partially consistent with existing data, though they also differ in some important ways. Recently, in a sample of 36 female chimpanzees, Lacreuse et al. (2014) reported age-related changes in response to gaze following, with older subjects performing more poorly. The results reported here are largely consistent with this finding, though in a much larger sample of chimpanzees that also included males. The inclusion of males was relevant in the present study because the findings showed that the decline in RJA performance occurred at an earlier age in males than it did in females. Life history and survival tables for chimpanzees have shown pronounced sex differences in life span, with males dying, on average, 7 years earlier than females (Dyke et al., 1995; Hill et al., 2001). Thus, the early decline in RJA performance abilities in males compared to females is consistent with the differences in relative life span and mortality between the sexes.

The evidence for age-related decline in the GM volume within the temporal lobe is, as far as we know, the first compelling evidence of age-related decline in cortical organization in chimpanzees. Several studies in chimpanzees that have examined age-related decline in total brain volume and weight, white matter volume, frontal lobe gray and white matter volume and hippocampal volume have failed to find age-related changes (Herndon et al., 1999; Sherwood et al., 2011; Chen et al., 2013). Therefore, the significant effect of age on GM volume was not anticipated and certainly contradicts previous findings in chimpanzees. However, the data presented here differ from these previous studies in two important ways that might explain the discrepancy in findings. First, we had a much larger sample size than previous studies (previous largest sample size was *n* = 97), particularly among males and individuals within the elderly group. Second, we focused on the temporal lobe GM in this study, a region that has not, until now, been explicitly quantified in previous studies examining age-related changes in cortical organization in chimpanzees.

In summary, individual differences in RJA performance was associated with in GM asymmetries in the p_STG in chimpanzees. These findings are consistent with evidence of the role of the posterior superior temporal lobe in the processing of socially relevant information in humans and monkeys. What factors or mechanisms underlie both variation in RJA performance and p_STG asymmetries are not clear from this study, but the findings indicate that additional consideration and investigation are warranted. We would further add that this study focused only on anatomy, but examining the functional role of the p_STG in relation to RJA performance should be explored in future studies as a means of understanding the ontogenetic and phylogenetic factors that underlie the perception of socially relevant communicative cues in primates, including humans.

## Conflict of Interest Statement

The authors declare that the research was conducted in the absence of any commercial or financial relationships that could be construed as a potential conflict of interest.
